# The Predictive Value of Proportional Evaluation Based on the Metabolic Activity of Cervical Lymph Nodes on PET/CT Imaging in Patients with Larynx Cancer

**DOI:** 10.4274/mirt.galenos.2019.92300

**Published:** 2020-04-29

**Authors:** Sevda Sağlampınar Karyağar, Savaş Karyağar, Osman Güven, Onur Üstün, Yavuz Atar, Yavuz Uyar

**Affiliations:** 1 University of Health Sciences Turkey, İstanbul Okmeydanı Training and Research Hospital, Clinic of Nuclear Medicine, İstanbul, Turkey; 2 University of Health Sciences Turkey, İstanbul Okmeydanı Training and Research Hospital, Clinic of Otorhinolaryngology, İstanbul, Turkey

**Keywords:** Cervical lymph node, metabolic activity, larynx cancer, positron emission tomography/computed tomography

## Abstract

**Objectives:**

We aimed to evaluate the proportional values of maximum standardized uptake value (SUV_max_) for cervical lymph nodes on ^18^F-fluorodeoxyglucose (FDG) positron emission tomography/computed tomography (PET/CT) for prediction of the presence of metastasis in patients with larynx squamous cell cancer (LSCC).

**Methods:**

This retrospective study involved 43 patients with LSCC. All patients underwent resection of the primary tumor and neck dissection within 4 weeks after undergoing ^18^F-FDG PET/CT examinations. Receiver operating characteristic (ROC) analysis was performed to evaluate the lymph node SUV_max_/primary tumor SUV_max_ (SUV_max_LN/SUV_max_PT), lymph node SUV_max_/aortic SUV_max_ (SUV_max_LN/SUV_max_A), and lymph node SUV_max_/liver SUV_max_ (SUV_max_LN/ SUV_max_L) ratios for diagnosis of lymph node metastasis.

**Results:**

SUV_max_LN/SUV_max_A, SUV_max_LN/SUV_max_L, and SUV_max_LN/SUV_max_PT rates were significantly higher in metastatic lymph nodes compared to non-metastatic nodes. ROC analysis for metastasis showed that the cut-off thresholds were 3.87 for SUV_max_LN; 1.78 for SUV_max_LN /SUV_max_A; 1.08 for SUV_max_LN/SUV_max_L; and 0.36 for SUV_max_LN/SUV_max_PT. The diagnostic sensitivity, specificity and AUC were 83.7%, 77%, 0.856 for SUV_max_LN; 79.7%, 84%, 1.78 for SUV_max_LN/SUV_max_A; 84.1%, 76%, 0.833 for SUV_max_LN/SUV_max_L; and 53.6%, 76%, 0.666 for SUV_max_LN/SUV_max_PT, respectively.

**Conclusion:**

SUV_max_LN/SUV_max_A, SUV_max_LN/SUV_max_L, and SUV_max_LN/SUV_max_PT ratios can be safely used for diagnosis of cervical lymph node metastasis in patients with LSCC.

## Abstract (tr)

**Amaç:**

^18^F-florodeoksiglikoz (FDG) pozitron emisyon tomografisi/bilgisayarlı tomografide (PET/BT) servikal lenf nodlarının maksimum standardize uptake değerine (SUV_maks_) dayalı oransal değerlerin skuamöz hücreli larenks kanserli (LSHK) hastalarda metastaz varlığını tahmin etmedeki gücünü değerlendirmeyi amaçladık.

**Yöntem:**

Bu retrospektif çalışmaya LSHK olan 43 hasta alındı. Tüm hastalara ^18^F-FDG PET/BT görüntülemeden sonraki 4 hafta içinde primer tümör rezeksiyonu ve boyun diseksiyonu yapıldı. Metastatik lenf nodlarının tanısı açısından lenf nodu SUV_maks_/primer tümör SUV_maks_ (SUV_maks_LN/SUV_maks_PT), lenf nodu SUV_maks_/aortik SUV_maks_ (SUV_maks_LN/SUV_maks_A), lenf nodu SUV_maks_/karaciğer SUV_maks_ (SUV_maks_LN/SUV_maks_L) oranları alıcı işletim karakteristiği (ROC) analizi yapılarak değerlendirildi.

**Bulgular:**

SUV_maks_LN/SUV_maks_A, SUV_maks_LN/SUV_maks_L, SUV_maks_LN/SUV_maks_PT oranları metastatik lenf nodlarında metastatik olmayanlara göre anlamlı derecede yüksek idi. ROC analizinde metastaz tespiti açısından eşik değerler, SUV_max_LN için 3,87; SUV_maks_LN/SUV_maks_A için 1,78; SUV_maks_LN/SUV_maks_L için 1,08; SUV_maks_LN/SUVmaxPT için 0,36 olarak bulundu. Tanısal duyarlılık, özgüllük ve AUC değerleri SUV_maks_LN, SUV_maks_LN/SUV_maks_A, SUV_maks_LN/SUV_maks_L, SUV_maks_LN/SUV_maks_PT için sırasıyla %83,7, %77, 0,856; %79,7, %84, 1,78; %84,1, %76, 0,833; %53,6, %76, 0,666 idi.

**Sonuç:**

SUV_maks_LN/SUV_maks_A, SUV_maks_LN/SUV_maks_L, SUV_maks_LN/SUV_maks_PT oranları, LSHK hastalarında metastatik servikal lenf nodlarının tanısında güvenle kullanılabilir.

## Introduction

^18^F-fluorodeoxyglucose (FDG) positron emission tomography/computed tomography (PET/CT) is being increasingly carried out to determine the stage and localization of metastatic disease in patients with larynx squamous cell cancer (LSCC). The correct diagnosis of metastatic cervical lymph nodes is important in terms of determining prognosis and providing adequate treatment. In clinical practice, CT and/or magnetic resonance imaging (MRI) are generally recommended for the assessment of tumor extension and cervical lymph node involvement (1).

Several studies in the literature reported that ^18^F-FDG PET/CT had reliable diagnostic value for a depiction of lymph node metastasis of head and neck squamous cell (HNSCC) compared with conventional CT/MRI (2,3).Meta-analyses of ^18^F-FDG PET/CT showed that the pooled per-patient, per-neck-side, and per-neck-level sensitivities/specificities were 0.91/0.87, 0.84/0.83, and 0.80/0.96, respectively. Across 13 studies (3460 neck levels) for which per-neck-level data were available, sensitivity and specificity were 0.84/0.96 respectively for ^18^F-FDG PET/CT and 0.63/0.96 for conventional imaging (CT and MRI), respectively (4).

Cut-off value of 2.5 for the maximum standardized  uptake value (SUV_max_) is used commonly to differentiate between benign and malignant lesions on ^18^F-FDG PET/CT imaging. But many biological and technical factors can affect SUV_max _value, such as patient’s weight, blood glucose level, postinjection uptake time, respiratory motion, tumor behavior, lesion size, motion artefacts, variability of the scanner, image-reconstruction parameters and contrast agent used (5,6). These factors may affect SUV values by 5% to 50% and cause false negativity or positivity (7).

In the literature, there are many studies on proportional values such as SUV_max_LN/ SUV_max_PT, SUV_max_LN/SUV_max_A, and SUV_max_LN/SUV_max_L for the prediction of metastatic lymph nodes in various malignancies, mainly lung cancer and breast cancer. However, the number of studies about LSCC is limited (8,9,10,11,12). In this study, we aimed to evaluate the proportional values of the SUV_max_ for cervical lymph nodes on ^18^F-FDG PET/CT imaging for the prediction of the presence of metastasis in patients with LSCC.

## Materials and Methods

This retrospective study involved 43 patients (42 men,1 woman; mean age=60.20±8.12 years, range=46-83) with LSCC.

The following criteria were defined for patient selection:

- Patients with diagnosis ofprimary LSCC made by a biopsy.

- Patients who did not undergo any treatment for LSCC before ^18^F-FDG PET/CT imaging and had no secondary malignancy.

- Patients who did not undergo any diagnostic excisional or incisional cervical lymph node biopsy for metastasis evaluation before ^18^F-FDG PET/CT imaging.

- Patients who underwent resection of the primary tumor and neck dissection within 4 weeks after undergoing ^18^F-FDG PET/CT imaging.

^18^F-FDG PET/CT imaging was performed at our institution between November 2013 and January 2018. Ethics Committee Approval was obtained from Okmeydanı Training and Research Hospital Ethics Committee with the decision number “1066” and date “12.04.2018”. The written informed consent was obtained from all patients at the time of imaging.

^18^F-FDG PET/CT studies were carried out using an integrated PET/CT scanner, which consisted of a full-ring HI-REZ LSO PET and a 6-slice CT (Siemens Biograph 6; Siemens, Chicago, USA). All patients were instructed to fast for at least 6 h before the ^18^F-FDG injection. Blood glucose levels were measured before the study and the injection was given only when the blood glucose levels were below 11.11 mmol/L. The patients were injected with 370 to 555 MBq ^18^F-FDG, according to body weight. After 60 minutes of waiting on a semireclined relaxed chair, the patients were imaged using an integrated PET/CT scanner. The CT portion of the study was performed without injection of intravenous contrast medium to define anatomical landmarks and attenuate correction on PET images. CT was acquired first with the following parameters: 50 mAs, 140 kV, and 5 mm section thickness. Whole-body CT was performed in a craniocaudal direction. PET images were acquired in a three-dimensional mode, from the vertex to mid-thigh, with six to eight bed positions of 3 min each, and PET data were collected in a caudocranial direction. Image reconstruction used “ordered subsets expectation maximization” algorithm of 2 iterations and 8 subsets. Image analysis was carried out on the Esoft multimodality computer platform (Siemens Medical Solutions, Erlangen, Germany). ^18^F-FDG PET/CT images were retrospectively interpreted by 3 experienced nuclear medicine physicians. The observers were blinded to the results of preoperative diagnostic imaging examinations such as MRI or ultrasonography and to the histopathological evidence of lymph node dissections. All cervical lymph nodes on CT which increased tracer uptake compared with background activity were accepted as metastatic. Semi-quantitative analysis of ^18^FDG uptake was performed, through creation of a region of interest (ROI) over the primary lesion and lymph nodes. SUV_max_ was also determined by manually placing a cylindrical ROI over the arcus aorta and right lobe of the liver. Lymph node SUV_max_ values were divided by the SUV_max_ of the primary tumour, arcus aorta (mediastinal blood pool) and liver to calculate the following:

- Lymph node SUV_max_/primary tumour SUV_max_ (SUV_max_LN/SUV_max_PT)

- Lymph node SUV_max_/aortic SUV_max_ (SUV_max_LN/SUV_max_A)

- Lymph node SUV_max_/Liver SUV_max_ (SUV_max_LN/SUV_max_L)

Operations were performed in our head and neck surgical clinic based on clinical and imaging findings. Modified radical neck dissection was performed in all patients. Lymph nodes and tumors were dissected from the specimens and stained with hematoxylin and eosin for histologic analysis. Serial histologic sections were used. We compared results of preoperative examinations using ^18^F-FDG PET/CT with those of the corresponding histopathologic examinations. If one lymph node showed increased uptake on ^18^F-FDG PET/CT images and had some findings on CT such as ≥10 mm diameter or round shape or hypoechogenicity or irregular margin or loss of fatty hilum findings and if histopathology showed lymph node with metastasis in the same neck level, this lymph node was accepted as a true positive finding for ^18^F-FDG PET/CT. If metastatic lymph node number on histopathology was lower than lymph node numbers that were accepted as metastatic on ^18^F-FDG PET/CT, the lymph node showing the lowest uptake was accepted as false positive. If metastatic lymph node number on histopathology was higher than lymph node number accepted as metastatic on ^18^F-FDG PET/CT, it was recorded as false-negative lymph node for ^18^F-FDG PET/CT.

### Statistical Analysis

While evaluating the findings of the study, IBM SPSS Statistics 22 program (IBM SPSS, Turkey) was used for statistical analysis. The normal distribution of the parameters was evaluated by the Shapiro-Wilk test and it was found that the parameters did not show normal distribution. The Mann-Whitney U test was used to compare the parameters between the two groups. Receiver operating characteristic (ROC) curve analysis was performed to identify the best cut-off value and to evaluate whether SUV_max_LN and also SUV_max_LN/SUV_max_PT, SUV_max_LN/SUV_max_A and SUV_max_LN/SUV_max_L ratios provided diagnosis of lymph node metastasis. For the calculation of sensitivity and specificity, the screening test was used. A p value <0.05 was accepted as statistically significant.

## Results

**Figure 1:**
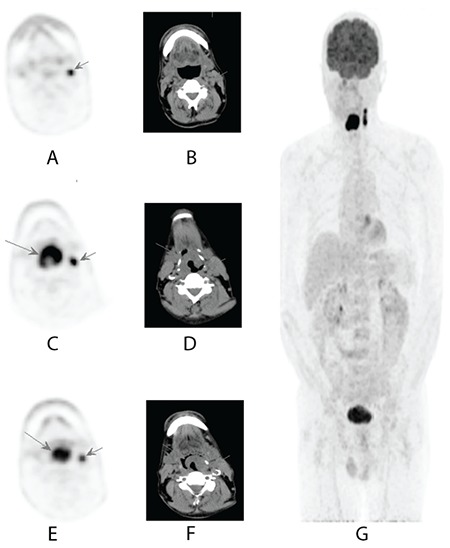
^18^F-FDG PET/CT with axial slice PET (A, C, E), axial slice CT (B, D, F) and whole-body PET (F) images of a 59-year-old patient with larynx squamous cell cancer. ^18^F-FDG PET/CT imaging showed primary laryngeal hypermetabolic lesion and hypermetabolic lymph nodes at left upper and middle jugular region compatible with metastasis. He underwent total laryngectomy and left modified neck dissection. Histopathological examination revealed three metastatic lymph nodes FDG: Fluorodeoxyglucose, PET: Positron emission tomography, CT: Computed tomography

Patients general characteristics are given in Table 1. Histopathological examination revealed 71 metastatic lymph nodes in 21 patients (mean=3.5; range=1-12). On ^18^F-FDG PET/CT imaging, 68 lymph nodes showing increased FDG uptake were evaluated by histopathological examination and metastasis was detected (Figure 1), but the histopathological examination of 26 lymph nodes showing ^18^F-FDG involvement showed no metastasis. There was no pathological involvement on ^18^F-FDG PET/CT in 3 lymph nodes with metastasis in 1 patient.

In patients with metastatic lymph nodes, the primary tumor SUV_max_ values were significantly higher than non-metastatic patients (26.76±10.43 vs 17.73±8.14; p=0.001). Mean SUV_max_LN, SUV_max_LN/SUV_max_Lratio, SUV_max_LN/SUV_max_A ratio, and SUV_max_LN/ SUV_max_PT ratio were significantly higher in patients with metastatic lymph nodes than non-metastatic nodes (Table 2).

**Figure 2:**
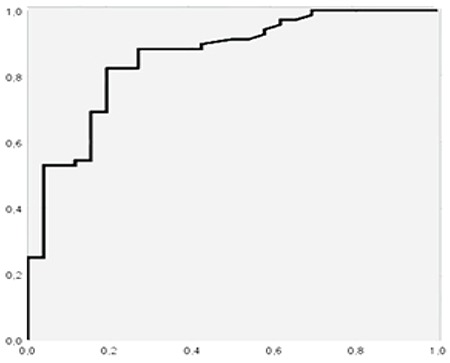
ROC curve for SUV_max_LN in the diagnosis of lymph node metastasis ROC: Receiver operating characteristic, SUV_max_: Maximum standardized uptake value, LN: Lymph node

**Figure 3:**
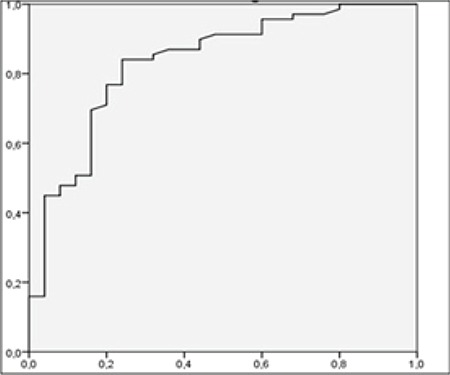
ROC curve for SUV_max_LN/SUV_max_L in the diagnosis of lymph node metastasis ROC: Receiver operating characteristic, SUV_max_: Maximum standardized uptake value, LN: Lymph node, L: Liver

**Figure 4:**
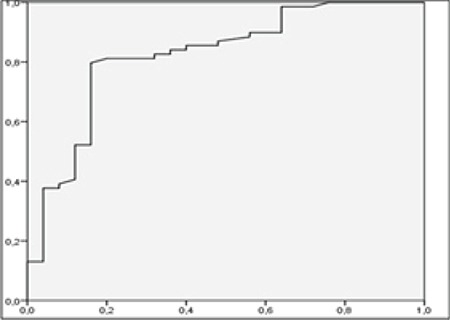
ROC curve for SUV_max_LN/SUV_max_A in the diagnosis of lymph node metastasis ROC: Receiver operating characteristic, SUV_max_: Maximum standardized uptake value, LN: Lymph node, A: Aortic

**Figure 5:**
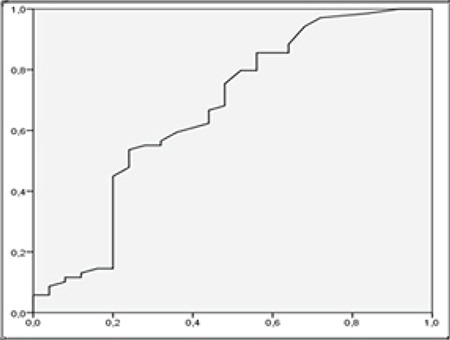
ROC curve for SUV_max_LN/SUV_max_PT in the diagnosis of lymph node metastasis ROC: Receiver operating characteristic, SUV_max_: Maximum standardized uptake value, LN: Lymph node, PT: Primary tumor

For diagnosis of lymph node metastasis with ROC analysis, the cut-off point for SUV_max_LN was 3.87 (AUC 0.856, p: 0.000) with the sensitivity of 83.7%, specificity 77%, positive predictive value (PPV) 89.1% and negative predictive value (NPV) 58% (Figure 2). The cut-off point for SUV_max_LN/SUV_max_L was 1.08 (AUC 0.833, p=0.000), with a sensitivity of 84.1%, specificity of 76%, PPV 90.6%, and NPV 63.3% (Figure 3). The cut-off point for SUV_max_LN/SUV_max_A was 1.78 (AUC 0.822, p=0.000) for the diagnosis of lymph node metastasis. The sensitivity of this value was 79.7%, specificity was 84%, PPV was 93.2%, and NPV was 60% (Figure 4). The cut-off point for SUV_max_LN/SUV_max_PT was 0.36 (AUC 0.666, p: 0.014) for the diagnosis of lymph node metastasis, with a sensitivity of 53.6%, specificity 76%, PPV 86.1%, and NPV 37.3% (Figure 5). When SUV_max_LN>2.5 was taken as a criterion for the detection of metastatic lymph nodes, the sensitivity was 95.7%, the specificity was 26.9%, PPV was 78%, and NPV was 70%.

## Discussion

Quantitative data obtained using ^18^F-FDG PET/CT can be useful for lymph node assessment in addition to visual evaluation. In general practice, if a lymph node shows ^18^F-FDG uptake and SUV_max_ value is more than 2.5, it is more likely to be malignant (13). But, SUV_max_ value does not have sufficient diagnostic capability to detect metastatic lymph nodes in head and neck cancer. In our study, if SUV_max_ >2.5 was taken as the criterion for detection of metastatic lymph node, the sensitivity would be 95.7% and the specificity would be 26.9%. To increase diagnostic accuracy for detecting metastatic lymph nodes, different lymph node SUVmax cut-off value and proportional ratios reproduced from lymph node SUVmax are used ( 12,14,15,16,17).

In our study, the mean SUV_max_ values, SUV_max_LN/SUV_max_L ratio, SUV_max_LN/ SUV_max_A ratio, and SUV_max_LN/SUV_max_PT ratio of metastatic lymph nodes were significantly higher than non-metastatic lymph nodes. In the ROC analysis, the cut-off point for SUV_max_LN was 3.87 for the diagnosis of lymph node metastasis, with a sensitivity of 83.7% and specificity of 77%. A study by Marshall et al. (16) included 114 patients with head and neck cancer and found that SUV_max_ cut-off was 3.9 and that yielded a sensitivity of 85% and specificity of 73%. Suenaga et al. (17) used a SUV_max_ cut-off value of 3.65 and found that sensitivity, specificity, and accuracy of ^18^F-FDG PET/CT on a level by level basis were 72.9, 96.8, and 92.1%, while sensitivity, specificity, and accuracy of CT were 52.9, 98.6, and 89.6%, respectively (17).

In our study, cut-off values for SUV_max_LN/SUV_max_L and SUV_max_LN/ SUV_max_A were 1.08 and 1.78, respectively. The sensitivity and specificity for these cut-off values were 84.1%-76% and 79.7%-84%, respectively. These proportional values were proven to have higher AUC compared to SUV_max_LN/SUV_max_PT ratio and have high diagnostic power for the diagnosis of metastatic lymph node. We think that this situation is due to the variance in SUV_max_ of the primary tumor due to the size or histopathological features of the tumor. The specificity of all 3 proportional values we examined in our patient group was higher when SUV_max_ >2.5 criterion was used. Lim et al. (12) studied 74 patients with HNSCC and found that nodal SUV_max_ ≥3.16 yielded a sensitivity of 74.4% and specificity of 84.9% in detecting metastatic nodes and also that nodal SUV_max_LN/SUV_max_L ratio ≥0.90 yielded a sensitivity of 74.1% and specificity of 93.4%.

### Study Limitations

There are some limitations of the present study. It was a retrospective study with a limited number of patients. As the study was retrospective, there were technical impediments to the matching of the lymph nodes detected on ^18^F-FDG PET/CT imaging and histopathological examination. Because the precise spatial correlation between ^18^F-FDG PET/CT and histopathology was impossible and one to one matching between them showed increased uptake on ^18^F-FDG PET/CT and metastatic lymph nodes on histopathologic evaluation could not be attributed due to the retrospective study design; analysis of results of the study should use this model. Therefore, there is a need for prospective studies with larger samples.

## Conclusion

SUV_max_LN/SUV_max_A, SUV_max_LN/SUV_max_L, and SUV_max_LN/SUV_max_PT ratios can be used safely for diagnostic evaluation of metastasis in cervical lymph nodes on ^18^F-FDG PET/CT imaging in patients with LSCC.

## Figures and Tables

**Table 1:**
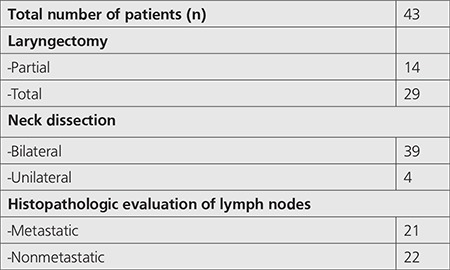
General characteristics of patients

**Table 2:**
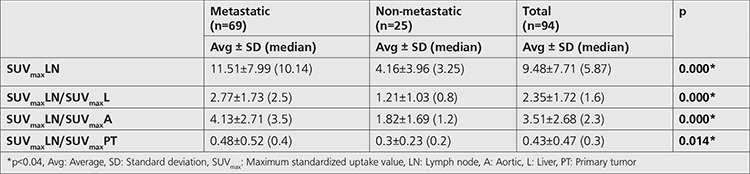
Diagnostic performance of the quantitative metabolic parameters
